# Non-human primate orthologues of TMPRSS2 cleave and activate the influenza virus hemagglutinin

**DOI:** 10.1371/journal.pone.0176597

**Published:** 2017-05-11

**Authors:** Pawel Zmora, Paulina Molau-Blazejewska, Stephanie Bertram, Kerstin Walendy-Gnirß, Inga Nehlmeier, Anika Hartleib, Anna-Sophie Moldenhauer, Sebastian Konzok, Susann Dehmel, Katherina Sewald, Constantin Brinkmann, Christoph Curths, Sascha Knauf, Jens Gruber, Kerstin Mätz-Rensing, Franziska Dahlmann, Armin Braun, Stefan Pöhlmann

**Affiliations:** 1 Infection Biology Unit, German Primate Center, Leibniz-Institute Kellnerweg 4, Göttingen, Germany; 2 Division Preclinical Pharmacology and In Vitro Toxicology, Fraunhofer Institute for Toxicology and Experimental Medicine; Biomedical Research in Endstage and Obstructive Lung Disease Hannover (BREATH), Member of the German Center for Lung Research, Nikolai-Fuchs-Strasse 1, Hannover, Germany; 3 Pathology Unit, German Primate Center, Leibniz-Institute, Kellnerweg 4, Göttingen, Germany; 4 Workgroup Neglected Tropical Diseases, German Primate Center, Leibniz-Institute, Kellnerweg 4, Göttingen, Germany; 5 Primate Genetics Laboratory, Junior Research Group "Medical RNA Biology," German Primate Center, Leibniz-Institute, Göttingen, Germany; CEA, FRANCE

## Abstract

The cellular serine protease TMPRSS2, a member of the type II transmembrane serine protease (TTSP) family, cleaves and activates the hemagglutinin of influenza A viruses (FLUAV) in cell culture and is essential for spread of diverse FLUAV in mice. Non-human primates (NHP), in particular rhesus and cynomolgus macaques, serve as animal models for influenza and experimental FLUAV infection of common marmosets has recently also been reported. However, it is currently unknown whether the NHP orthologues of human TMPRSS2 cleave and activate FLUAV hemagglutinin and contribute to viral spread in respiratory tissue. Here, we cloned and functionally analyzed the macaque and marmoset orthologues of human TMPRSS2. In addition, we analyzed the macaque orthologues of human TMPRSS4 and HAT, which also belong to the TTSP family. We found that all NHP orthologues of human TMPRSS2, TMPRSS4 and HAT cleave and activate HA upon directed expression and provide evidence that endogenous TMPRSS2 is expressed in the respiratory epithelium of rhesus macaques. Finally, we demonstrate that a serine protease inhibitor active against TMPRSS2 suppresses FLUAV spread in precision-cut lung slices of human, macaque and marmoset origin. These results indicate that FLUAV depends on serine protease activity for spread in diverse NHP and in humans. Moreover, our findings suggest that macaques and marmosets may serve as models to study FLUAV activation by TMPRSS2 in human patients.

## Introduction

Influenza A virus (FLUAV) infection is a major source of global morbidity and mortality. The annual influenza epidemics claim up to 500,000 lives, with children, the elderly and patients with compromised immune system being mainly affected. Moreover, intermittent influenza pandemics might have even more dramatic consequences [1–3]. Currently available vaccines protect only against seasonal influenza and need to be reformulated annually while antivirals are the only defense against pandemic FLUAV. Antiviral drugs targeting the viral proteins neuraminidase (NA) and M2 are available but their antiviral activity can be compromised by resistance development [4]. In particular, the use of M2 inhibitors is not recommended due to high frequency of viral resistance. Therefore, novel antiviral strategies are required to provide protection against FLUAV. Host cell proteins essential for viral spread but dispensable for survival of the cell are attractive targets for novel approaches to antiviral intervention, since their blockade might be associated with a high barrier against viral resistance [5].

The viral surface protein hemagglutinin (HA) mediates binding and entry of FLUAV into host cells and is the central target for neutralizing antibodies. The HA protein is synthesized as an inactive precursor protein in the constitutive secretory pathway of infected cells and depends on cleavage by host cell proteases to become active [6,7]. Cleavage-activation is essential for viral infectivity [8–10]. As a consequence, the responsible proteases are potential targets for antiviral intervention. It has been speculated that diverse secreted proteases can cleave and activate HA in the infected host [11]. However, recent studies demonstrate that a single membrane-associated enzyme with unknown physiological function, the serine protease TMPRSS2, cleaves and activates FLUAV in cell culture and is essential for spread of diverse FLUAV in mice [12–18]. Moreover, polymorphisms in *tmprss2* were found to be associated with influenza severity in human patients [19], suggesting that FLUAV might also hijack TMPRSS2 for spread in the human host. However, direct proof for this hypothesis remains to be provided. Moreover, 17 TTSPs were identified in humans and several of them, including TMPRSS4 and HAT, were shown to be expressed in lung and to activate HA upon directed expression in cell culture [20–27]. The potential contribution of these proteases to viral spread in the host is largely unclear, although a recent study demonstrated that H3N2 viruses might be able to exploit TMPRSS4 jointly with TMPRSS2 for spread in mice [28].

The experimental infection of macaques with FLUAV results in viral spread and influenza symptoms, which are usually not as pronounced as those observed in human influenza patients [29,30]. Nevertheless, infection of macaques with highly pathogenic FLUAV, the pandemic H1N1 virus of 1918 and H5N1 avian influenza viruses, induces severe disease and the macaque model has been successfully used for vaccine-, treatment- and pathogenesis-studies [31–33]. Based on these studies, it has been suggested that the macaque model mirrors human physiology, development of pneumonia as well as cytokine and chemokine responses more closely than other animal models (mice, ferrets) [34]. Recently, experimental inoculation of common marmosets with FLUAV has also been described [30,35]. FLUAV spread and transmission has been observed in this model and the animals developed several but not all symptoms seen in human patients [35]. Collectively, NHP are valuable models for influenza and, due to their high genetic similarity to humans, these animals may be best suited to study dependence of FLUAV on host cell factors for spread and pathogenesis. However, it is currently unknown whether the NHP orthologues of TMPRSS2 and related proteases activate FLUAV and whether serine protease activity is required for FLUAV spread in the respiratory epithelium of primates.

Here, we show that TMPRSS2, TMPRSS4 and HAT of NHP origin can cleave and activate FLUAV HA of the H1 subtype and that TMPRSS2 is expressed in macaque respiratory epithelium. Moreover, we demonstrate that spread of FLUAV (H1N1 subtype) in human, macaque and marmoset lung tissue can be blocked by a serine protease inhibitor active against TMPRSS2.

## Materials and methods

### Plasmids, cells and viruses

Expression plasmids for H1 HA (A/South Carolina/1/1918), human TMPRSS2, TMPRSS4 and HAT were described previously [12,21]. For construction of the plasmids containing NHP orthologues of human proteases, cDNA was prepared from NHP lung tissue and protease sequences PCR amplified and cloned as indicated in Table 1. The human embryonal kidney (HEK) 293T (ACC-635, Leibniz Institute DSMZ–German Collection of Microorganisms and Cell Cultures) cells were used for the transfection and infection experiments and were propagated in Dulbecco's modified Eagle's medium (DMEM, PAN). MDCK cells (ATCC CRL-2936, kindly provided by K. Schugart, HZI, Braunschweig) were used for the focus formation assay and were grown in minimum essential medium (MEM, Gibco). All media were supplemented with fetal bovine serum (10%, FBS Biochrome), penicillin, and streptomycin. The cells were maintained in a humidified atmosphere containing 5% CO_2_. The FLUAV A/PR/8/34 (H1N1) and A/Hamburg/04/2009 (H1N1) were propagated in the chorioallantoic cavities of 10-days-old embryonated hen eggs (Valo BioMedia, Germany) for 48 h at 37°C, as previously described [36]. Viral titers were determined using the focus formation assay, which was performed exactly as previously described [22,36].

**Table 1:** Zmora *et al*.

NHP	TTSP	Oligo	Enzyme	Sequence (5‘→3‘)
Rhesus macaque	*tmprss2*	Out 5’	-	GCTCGGCAGGTCATACTGAACATTCCAGATACC
		Out 3’	-	CGGGGAAGCAAAACCAGCCGCTTTGTTTTCTCG
		In 5’	*EcoRI*	CGGCGAATTCCACCATGGCTTTGAACTCAGGGTCACCGC
		In 3’	*NheI*	GCGGCTAGCTTAGTCATCTGCCCTCATTTGTCG
	*tmprss4*	Out 5’	-	CCTCCTGCTGCCTTGGGGTGACAATCTCAC
		Out 3’	-	GGGGATCCCCAGGTGGGCAGGGC
		In 5’	*ClaI*	CCGCCATCGATCCACCATGGATCCTGACAGTGATCAACCTC
		In 3’	*XhoI*	CCGCCTCGAGTTACAGCTCAGCCTTGCGGACATTGTAG
	*hat*	Out 5’	-	CCAGCTACACAGGAATACAGGACTTTGAGTGG
		Out 3’	-	GTAAAGCTTTGGAATTTAAGACAGGCACACCCGC
		In 5’	*EcoRI*	CGGGCGAATTCCACCATG AGAGCTCATGTTGTCAAACTGAG
		In 3’	*XhoI*	CCGCCTCGAGCTAGATCCCAGTTCGTTGCCTAATCCAG
Cynomolgus macaque	*tmprss2*	Out 5’	-	GCTCGGCAGGTCATACTGAACATTCCAGATACC
		Out 3’	-	CGGGGAAGCAAAACCAGCCGCTTTGTTTTCTCG
		In 5’	*EcoRI*	CGGCGAATTCCACCATGGCTTTGAACTCAGGGTCACCGC
		In 3’	*NheI*	GCGGCTAGCTTAGTCATCTGCCCTCATTTGTCG
	*tmprss4*	Out 5’	-	CCTCCTGCTGCCTTGGGGTGACAATCTCAGC
		Out 3’	-	GGGGATCCCCAGGTGGGCAGGGC
		In 5’	*ClaI*	CCGCCATCGATCCACCATGGATCCTGACAGTGATCAACCTC
		In 3’	XhoI	CCGCCTCGAGTTACAGCTCAGCCTTGCGGACATTGTAG
	*hat*	Out 5’	-	ATTTGAGTGGGAATCTCAAAGCAGTTGAGTAGGC
		Out 3’	-	GTAAAGCTTTGGAATTTAAGACAGGCACACCCGC
		In 5’	*EcoRI*	CGGGCGAATTCCACCATGTATAGGCCAGCACGTGTACCATC
		In 3’	*XhoI*	CCGCCTCGAGCTAGATCCCAGTTCGTTGCCTAATCCAG
Common Marmoset	*tmprss2*	Out 5’	-	CCTCTAACTGGTGCGATGG
		Out 3’	-	GGATACACCATGTAGCCATTGG
		In 5’	*EcoRI*	GCGGAATTCTTAACCATGGCTTTGAACTCAGGGTCAC
		In 3’	*XhoI*	CGCTCGAGTCACACGTGTATCCTCATTTGTTGGTAAATC

### Bioinformatic analysis

Nucleotide and amino acid sequences were analyzed using the nucleotide (nt) BLAST search option available via the server at NCBI (http://blast.ncbi.nlm.nih.gov/Blast.cgi). The alignments of the amino acid sequences of the human and NHP TTSPs were constructed using AlignX Vector NTI (ThermoFisher).

### Western blot analysis

To analyze the expression of human TMPRSS2, TMPRSS4 and HAT and their NHP orthologues, 293T cells were seeded into 6-well plates at a density of 2.8 x 10^5^ cells/well, cultivated overnight and then transfected with plasmids encoding the proteases equipped with an N-terminal myc tag, or transfected with empty plasmid (pCAGGS) as a control. For the analysis of HA cleavage, 293T cells seeded in 6-well plates were cotransfected with plasmids encoding H1 HA and proteases, or empty plasmid as control. At 48 h post transfection, the cells were washed with PBS, resuspended in 2x sodium dodecyl sulfate (SDS) loading buffer and then heated at 95°C for 30 min. Proteins were separated by SDS-polyacrylamide gel electrophoresis (PAGE) and blotted onto a nitrocellulose membrane (Hartenstein). Protease expression was detected using a mouse anti-myc antibody (Biomol) as the primary antibody and a horseradish peroxidase (HRP)-coupled antibody (Dianova) as the secondary antibody. The FLUAV HA cleavage was detected by staining with a goat anti-FLUAV polyclonal antibody (Millipore) and HRP-coupled anti-goat secondary antibody (Dianova). Expression of β-actin was detected with anti-β-actin antibody (Sigma Aldrich) and served as a loading control.

### Preparation of precision-cut lung slices

Preparation of precision-cut lung slices (PCLS) was performed as previously described [37,38]. Briefly, lung lobes (human) or lung (NHP) were cannulated and filled with prewarmed 1.5% agarose (low gelling temperature agarose, Sigma-Aldrich) in cell culture medium (MEM HEPES modification, Sigma-Aldrich) and allowed to solidify on ice (human, macaque) or in ice-cold PBS (common marmoset). Lung lobes were separated, cut, and punched to obtain lung tissue containing central airways. PCLS with a diameter of 8 mm were generated employing a Live Tissue Microtome (Krumdieck tissue slicer, Alabama Research and Development). PCLS were transferred into DMEM/F-12, HEPES, no phenol red medium (Gibco) supplemented with 0.2% BSA and P/S medium (PCLS medium) in 24 well plates on 37°C and washed for three times every 30 minutes.

### Infection experiments

For infection experiments, 293T cells were seeded in 6-well plates at a density of 2.8 x 10^5^ cells/well cultivated overnight and then transfected with plasmids encoding proteases or empty plasmid as control. At 24 h post transfection, the cells were infected with FLUAV A/PR/8/34 at an MOI 0.01. For this, the cells were gently washed with pre-warmed Dulbecco's Phosphate-Buffered Saline (DPBS) supplemented with Ca^2+^ and Mg^2+^ (PAN) and then incubated with 500 μl of DPBS with Ca^2+^ and Mg^2+^ containing FLUAV for 1 h at 37°C in a humidified atmosphere. Next, the cells were gently washed with DPBS, and fresh infection medium (DMEM supplemented with 0.2% BSA, penicillin and streptomycin) was added. To analyze virus spread, culture supernatants were collected at 48 h post infection and the amount of infectious units within the supernatants was determined by focus formation assay, as described previously [39].

For the infection of PCLS, the cultures were incubated overnight in fresh PCLS medium, gently washed and infected for 1 h with 3 x 10^4^ ffu of FLUAV A/Hamburg/04/2009 (human PCLS) or A/PR/8/34 (NHP PCLS) diluted in 250 μl of PCLS medium. Afterwards, the slices were gently washed with pre-warmed DPBS with Ca^2+^ and Mg^2+^ (PAN) and 500 μl of PCLS medium containing 0, 1, 10 or 100 μM camostat mesylate (Sigma Aldrich) was added. The cultures were incubated for 48 h in 5% CO_2_ at 37°C and then the supernatants were collected and the viral titers were determined using the focus formation assay. In addition, the cultures were tested for cytotoxic effects, employing Cytotoxicity Detection Kit PLUS LDH (Roche). For immunohistochemistry, the PCLS were washed three times for 30 min with DPBS and fixed with 4% PFA.

#### Immunohistochemistry

Immunohistochemistry (IHC) was performed using the DAB-Map-Kit (Roche) on 2–4 μm thick sections of formalin-fixed paraffin embedded PCLS after cell conditioning with EDTA antigen retrieval solution pH 8.4. Sections were loaded into the DiscoveryXT autostainer (Roche), deparaffinized, rehydrated and incubated with the anti-TMPRSS2 antibody [EPR3861] (Abcam) in a dilution of 1 in 1,000 for 32 minutes at 37°C followed by incubation with the secondary antibody (universal secondary antibody, Ventana, Roche) for 24 minutes. Diaminobenzidin (Ventana/View DAB, Detection Kit, Roche) was applied as chromagen according to the supplier´s instructions. Slides were counterstained with hematoxylin and bluing reagent before examination by light microscopy. Tissue sections from small intestine of healthy rhesus macaques served as positive control samples. Negative control staining was performed by omission of the primary antibody for immunohistochemistry.

### Ethics statement

NHP lung: All animal work was conducted according to relevant national and international guidelines, in particular EU directive 2010/63/EU and the German animal protection law. The Animal Welfare and Ethics Committee of the German Primate Center approved the entire study and use of lung material from NHPs. Lungs originated from rhesus macaques (*Macaca mulatta*), cynomologus macaques (*Macaca fascicularis*) and common marmosets (*Callithrix jacchus*) of the German Primate Center. Animals were euthanized by an authorized veterinarian for animal welfare reasons, i.e. suffering from severe trauma after aggressive group conflicts, automutilation or marmoset wasting syndrome. Before euthanasia, animals were deeply anaesthetized using a combination of ketamine (Ketavet®, Pfizer) and xylazine (Rompun®, Bayer). Macaques were then euthanized by a lethal dose of sodium pentobarbital (Narcoren®, Merial GmbH) given intravenously. Marmosets were euthanized by a lethal dose of sodium pentobarbital (Narcoren®, Merial GmbH) given intracardially. Only animals with a healthy respiratory system were included in the study.

Human lung: Human lung explants were obtained from patients who underwent lung resection for cancer or pulmonary hypertension at hospitals in Hanover, Germany (Hannover Medical School, Klinikum Siloah/ Nordstadt). All patients gave written consent. The experimentation with human lung tissue was approved by the ethics committee of the Hannover Medical School and Klinikum Region Hannover. Only lung tissue containing no tumors as qualified by medical pathologists was used for the experiments. Tissue was processed immediately on the day of resection.

Hen eggs: Hen eggs were purchased from Valo BioMedia, Germany. At 48 h after inoculation with virus, the eggs were euthanized by an overnight incubation at 4°C and the allantoic fluid was harvested. According to "§ 14 TierSchVersV–Geltung für Tiere in einem frühen Entwicklungsstadium" experiments conducted before the last third of the development of the animal commences do not require approval.

## Results

### Robust expression of non-human primate orthologues of TMPRSS2, TMPRSS4 and HAT in transfected cells

**Fig 1:**
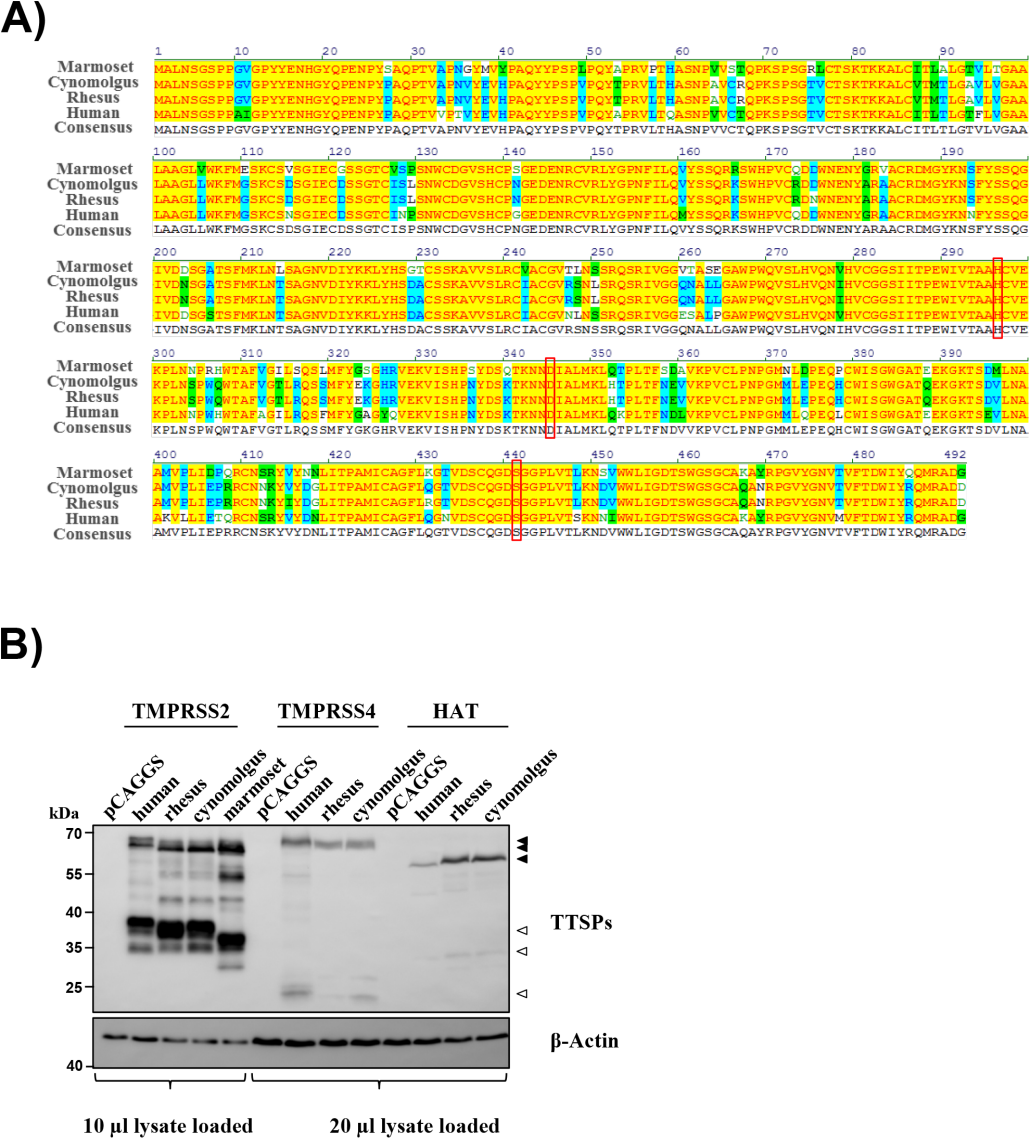
TMPRSS2, TMPRSS4 and HAT are conserved between humans and non-human primates. (A) Amino acid sequence alignment of human (NP_005647.3), rhesus macaque (XP_014988331.1), cynomolgus macaque (XP_015302312.1) and common marmoset (XP_008984973.1) TMPRSS2. Protein alignment was performed by using Vector NTI AlignX. Colors indicate amino acid identity (yellow), conservation (blue) and similarity (green). The catalytic triad is boxed. (B) For analysis of protease expression, 293T cells were transfected with plasmids encoding TMPRSS2, TMPRSS4 or HAT of the indicated species and equipped with an N-terminal myc antigenic tag. Empty plasmid (pCAGGS) served as a negative control. Protease expression in cell lysates was detected via Western blotting with anti-myc antibody. Due to more prominent expression of TMPRSS2 relative to TMPRSS4 and HAT proteins, 10 μl of lysates from TMPRSS2 expressing cells and 20 μl of lysates from TMPRSS4 and HAT expressing cells were loaded for separation by SDS gel-electrophoresis. The expression of β-actin was determined as a loading control. Filled triangles indicate zymogen forms, while empty triangles highlight cleavage products resulting from autocatalytic activation. The results were confirmed in at least two separate experiments.

For the functional characterization of TMPRSS2 of rhesus and cynomolgus macaques as well as marmosets, we PCR-amplified the respective open reading frames from lung mRNA. To this end, we employed RT-PCR with oligonucleotides based on sequence information available in Genbank. Sequences encoding macaque TMPRSS4 and HAT were amplified and analyzed for comparison. All PCR products were cloned into plasmid pCAGGS and the encoded protein sequences were found to be identical to those deposited in Genbank (human TMPRSS2: NP_005647.3, rhesus macaque TMPRSS2: XP_014988331.1, cynomolgus macaque TMPRSS2: XP_015302312.1 and common marmoset TMPRSS2: XP_008984973.1; human TMPRSS4: NP_063947.1, rhesus macaque TMPRSS4: XP_001092969.3, cynomolgus macaque TMPRSS4: XP_015291266.1; human HAT: NP_004253.1, rhesus macaque HAT: XP_001109114.1, cynomolgus macaque HAT: XP_005555244.1). The NHP encoded TMPRSS2 orthologues shared 86.0 (common marmoset), 87.8 (rhesus) or 88.0% (cynomolgus) sequence identity with human TMPRSS2 on the amino acid level and encoded the catalytic triad required for protease activity (Fig 1A). Similarly, rhesus and cynomolgus HAT both shared 92.3% amino acid sequence identity with the human protein, while the NHP orthologues of TMPRSS4 were to 95.9% (rhesus) and 92.4% (cynomolgus) identical to their human counterpart (not shown).

We next asked if the proteases were expressed in transfected 293T cells. For this, all proteases were equipped with an N-terminal myc-antigenic tag and expression analyzed by Western blot. In general, expression of TMPRSS2 proteins was more robust than expression of TMPRSS4 and HAT proteins (Fig 1B). Human TMPRSS2 and its NHP orthologues were expressed at comparable levels and similar observations were made for TMPRSS4. In contrast, expression of human HAT was somewhat reduced compared to its macaque orthologues (Fig 1B). TTSPs are synthesized as inactivate precursors, zymogens, which undergo autocatalytic activation. Indeed, bands with molecular weights expected for the zymogen forms and activation products of TMPRSS2, TMPRSS4 and HAT were detected (Fig 1B), suggesting that these enzymes are active in 293T cells. Collectively, the NHP orthologues of TMPRSS2, TMPRSS4 and HAT share high sequence identity with their human counterparts and are expressed and activated in transfected cells.

### TMPRSS2, TMPRSS4 and HAT of non-human primate origin cleave and activate the influenza A virus hemagglutinin

**Fig 2:**
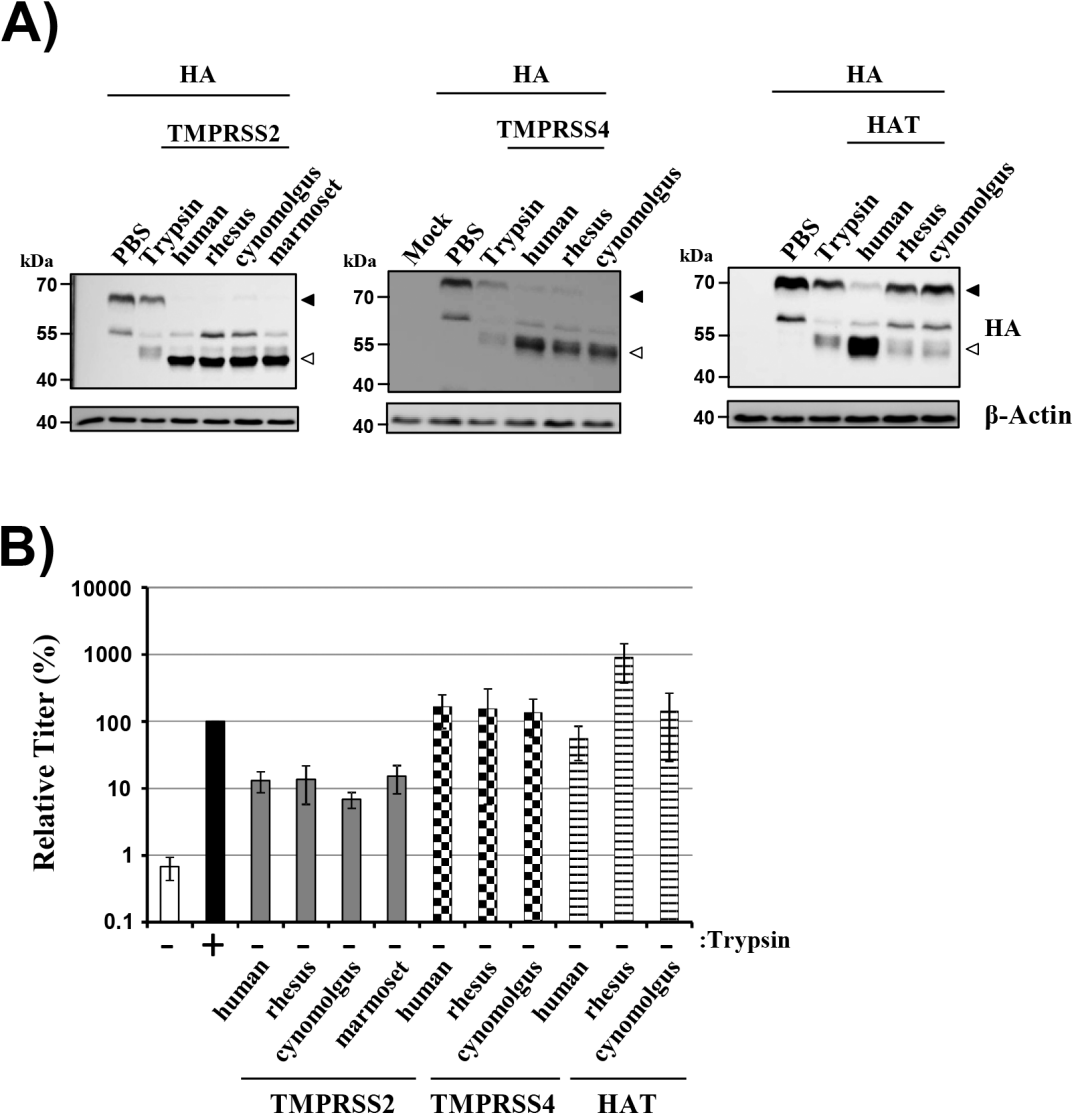
TMPRSS2, TMPRSS4 and HAT of non-human primate origin cleave and activate influenza virus hemagglutinin. (A) 293T cells were transiently cotransfected with plasmids encoding FLUAV HA of the 1918 H1N1 FLUAV and the indicated proteases or empty plasmid (pCAGGS). At 48 h post transfection the cells were treated with PBS or trypsin, and HA cleavage was determined by Western blotting. The HA precursor HA0 (filled triangle) and the surface unit HA1 (empty triangle) are indicated. The expression of β-actin was determined as a loading control. Similar results were obtained in three independent experiments. (B) The indicated proteases were expressed in 293T cells and the cells infected with FLUAV A/PR/8/34 (H1N1) at an MOI 0.01 and treated with either trypsin or PBS. At 48 h post infection, the virus titers were determined by focus formation assay. The average of three to five independent experiments is shown; error bars indicate standard error of the mean. Virus titers measured upon trypsin treatment were set as 100%.

We next investigated whether TMPRSS2, TMPRSS4 and HAT of NHP origin can cleave FLUAV-HA. Treatment of HA expressing cells with trypsin resulted in the cleavage of the HA precursor, HA0, as evidenced by the appearance of the HA1 subunit (Fig 2A), in keeping with expectations. Similarly, coexpression of human TMPRSS2 and TMPRSS4 as well as their NHP counterparts facilitated robust and comparable HA cleavage. Processing of HA by human HAT was also readily detectable but the macaque orthologues of this enzyme showed reduced HA cleavage (Fig 2A), despite their increased expression relative to the human enzyme. Finally, it is noteworthy that TMPRSS4/HAT and trypsin produced identical HA1 bands while the HA1 fragments generated by TMPRSS2 and trypsin differed in molecular weight (Fig 2A). These differences were previously reported and were found to be due to differential N-glycosylation of the HA1 fragments [12].

We next determined if HA cleavage results in activation. For this, the proteases were expressed in 293T cells, the cells infected with A/PR/8/34 and the infectious units present in culture supernatants at 48 h post infection were determined. Proteases endogenously expressed in 293T cells fail to activate HA [12,22] and therefore virus spread in the absence of trypsin was within background levels while spread in the presence of trypsin was efficient (Fig 2B). Expression of TMPRSS2 of human and NHP origin allowed robust spread in the absence of trypsin and similar observations were made for TMPRSS4 and HAT (Fig 2B). Thus, TMPRSS2, TMPRSS4 and HAT of NHP origin can cleave and activate HA.

### TMPRSS2 is expressed in macaque lung tissue and serine protease activity is required for FLUAV spread in non-human primate lung tissue

**Fig 3:**
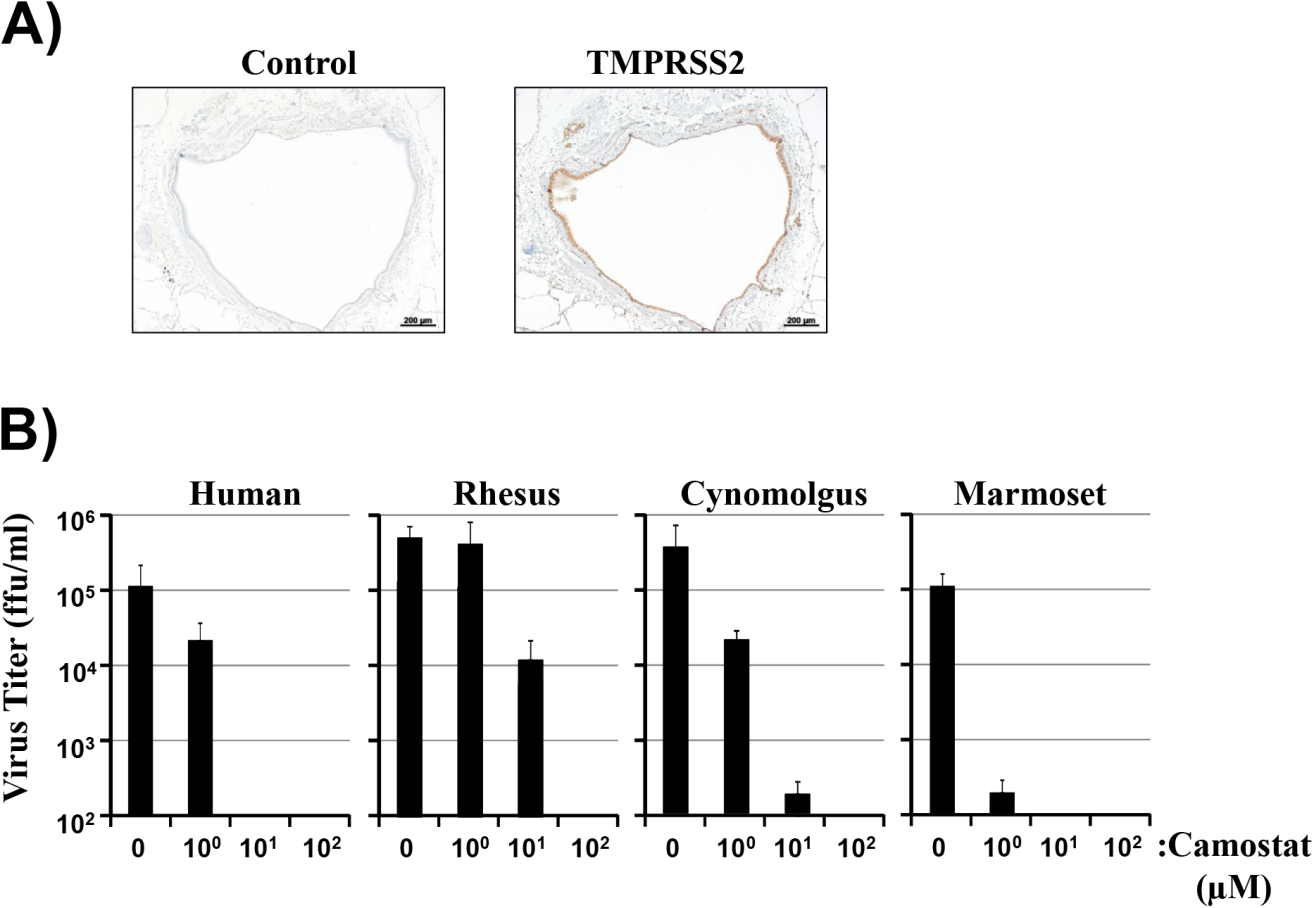
Serine protease activity is required for influenza A virus spread in respiratory epithelium of human and non-human primate origin. (A) Expression of TMPRSS2 in precision-cut lung slices (PCLS) of rhesus macaque origin was analyzed employing immunohistochemistry with an antibody raised against human TMPRSS2 which cross-reacts with the rhesus macaque orthologue. Hematoxylin was used for counterstaining. Omission of the primary antibody served as negative control. (B) Precision cut lung slices (PCLS) prepared from human, rhesus macaque, cynomolgus macaque or common marmoset lung were infected with 3 x 10^4^ ffu of FLUAV A/Hamburg/04/2009 (H1N1, human PCLS) or A/PR/8/34 (H1N1, rhesus, cynomolgus, marmoset PCLS) and treated with the indicated amounts of camostat mesylate. At 48 h post infection, the viral titers in the supernatants were tested using focus formation assay. The results of representative experiments performed with triplicate samples are shown. Error bars indicate standard deviations. Similar results were obtained in three to five independent experiments. ffu, focus forming units.

In the light of the pivotal role of TMPRSS2 in FLUAV spread in mice, we next examined whether TMPRSS2 protein is expressed in macaque lung tissue and whether an inhibitor of TMPRSS2 blocks viral spread in this tissue. For this, we employed PCLS of NHP origin. Immunohistochemistry conducted with an antibody raised against human TMPRSS2 but cross-reactive with the macaque orthologues revealed that TMPRSS2 (brown color) was robustly expressed in bronchiolar epithelial cells and subepithelial alveolar macrophages (Fig 3A). Camostat is a serine protease inhibitor active against TMPRSS2 [40] and afforded the opportunity to investigate whether FLUAV spread in human and non-human primate PCLS depends on serine protease activity. FLUAV spread in PCLS of human and NHP origin was generally efficient and susceptible to inhibition by camostat in the absence of cytotoxic effects (Fig 3B), as determined by Cytotoxicity Detection Kit PLUS LDH (not shown). However, subtle differences in the efficiency of FLUAV inhibition by camostat were noted. Thus, blockade of viral spread in common marmoset PCLS was most efficient, with 1 μM camostat reducing FLUAV spread to background levels, while this concentration of inhibitor had only modest inhibitory effects in PCLS of human and cynomolgus macaque origin and failed to block viral spread in rhesus macaque PCLS (Fig 3B). Thus, TMPRSS2 is expressed in macaque respiratory epithelium and serine protease activity is required for FLUAV spread in lung tissue of human, macaque and marmoset origin.

## Discussion

A constantly accumulating body of evidence suggests that TMPRSS2 is exploited by FLUAV for spread. Thus, directed and endogenous expression of TMPRSS2 in cell culture was shown to activate FLUAV HA [12,14,15] and the protease was found to be expressed in receptor-positive cells in the human aerodigestive tract [13]. Moreover, several studies reported that TMPRSS2 expression is required for spread and pathogenesis of diverse FLUAV in mice [16,18,41]. Furthermore, inactivation of the *tmprss2* gene in mice is compatible with normal development and homeostasis [42], indicating that TMPRSS2 is dispensable for cellular and organismic survival and thus an attractive drug target. However, the contribution of TMPRSS2 to viral spread in human and NHP is less well understood. Here, we show that the macaque and marmoset orthologues of TMPRSS2 cleave and activate FLUAV HA and that TMPRSS2 is expressed in rhesus macaque respiratory epithelium. Moreover, we demonstrate that FLUAV spread in human, macaque and marmoset respiratory epithelium can be blocked by a serine protease inhibitor active against TMPRSS2. These results suggest that macaques might be suitable to model TMPRSS2 usage by FLUAV in humans.

The members of the TTSP family show a conserved domain organization. The N-terminal domain is located in the cytoplasm and is followed by a transmembrane domain, a stem region and a protease domain [27]. The enzymes are synthesized as zymogens in the constitutive secretory pathway and can be auto-catalytically activated by cleavage between the stem region and the protease domain [27]. The enzymes encoded by *tmprss2* of NHP origin exhibited up to 88% amino acid sequence identity with their human counterparts, including the presence of a catalytic triad located in the protease domain that is essential for enzymatic activity, and similar observations were made for the NHP orthologous TMPRSS4 and HAT. Moreover, all tested proteases were readily expressed in transfected 293T cells and evidence for auto-activation in these cells was obtained. Therefore, it was not unexpected that all TMPRSS2, TMPRSS4 and HAT orthologues of NHP origin were able to cleave and activate HA. Whether the subtle differences in activation efficiency observed between marmoset TMPRSS2 and the other TMPRSS2 orthologues tested translate into differential HA activation in the infected host is at present unknown.

Our previous analysis demonstrated that TMPRSS2 is coexpressed with 2,6-linked sialic acid, the major receptor determinant of human FLUAV, in large parts of the human respiratory and gastrointestinal epithelium [13]. Expression was detected in human bronchiolar epithelium [13] and the present study indicates that substantial levels of TMPRSS2 are also produced in bronchiolar epithelial cells of rhesus macaques. However, potential differences in the cell type specificity of TMPRSS2 expression in human and NHP respiratory tissue can at present not be excluded. Thus, we previously detected TMPRSS2 protein mainly in type II pneumocytes within human tissue samples [13] and these cells can be targeted by FLUAV. In contrast, Matsuyama and colleagues found that TMPRSS2 was predominantly expressed in type I pneumocytes in healthy cynomolgus macaques, although in the context of severe acute respiratory syndrome coronavirus (SARS-CoV) infection, they detected viral antigen and TMPRSS2 protein in type II pneumocytes [43].

The findings that TMPRSS2 and related serine proteases activated FLUAV HA upon directed expression in cell culture and that endogenous TMPRSS2 was expressed in bronchiolar epithelium within PCLS triggered the question whether serine protease activity is required for FLUAV spread in human and NHP PCLS. We addressed this question employing the serine protease inhibitor camostat, since this compound was previously shown to block viral activation by TMPRSS2 in the absence of unwanted cytotoxicity [40], and since PCLS are not amenable to extensive manipulation, including transfection with siRNAs. Camostat markedly and dose-dependently reduced FLUAV spread in PCLS of human, macaque and marmoset origin without inducing cytotoxic effects, in keeping with recent data showing that camostat inhibits FLUAV replication and cytokine production in cultures of human tracheal epithelial cells [44]. Thus, FLUAV depends on the activity of a serine protease(s), potentially TMPRSS2, for spread in human and NHP respiratory tissue, although it should be stated that exclusively viruses of the H1N1 subtype were tested in the present study and it cannot be excluded that protease choice of other subtypes may differ.

Collectively, our results indicate that NHP orthologues of human TMPRSS2 and related proteases can activate HA and that macaques can serve as model to investigate protease-dependence of FLUAV spread.
